# Full versus half dose of antenatal betamethasone to prevent severe neonatal respiratory distress syndrome associated with preterm birth: study protocol for a randomised, multicenter, double blind, placebo-controlled, non-inferiority trial (BETADOSE)

**DOI:** 10.1186/s12884-019-2206-x

**Published:** 2019-02-12

**Authors:** Thomas Schmitz, Corinne Alberti, Moreno Ursino, Olivier Baud, Camille Aupiais, for the BETADOSE study group and the GROG (Groupe de Recherche en Gynécologie Obstétrique)

**Affiliations:** 1 Service de Gynécologie Obstétrique, Hôpital Robert Debré, Assistance Publique-Hôpitaux de Paris, 48 boulevard Sérurier, 75019 Paris, France; 2 Université Paris Diderot, Site Villemin, 10 avenue de Verdun, 75010 Paris, France; 3 Inserm, U1153, Epidemiology and Biostatistics Sorbonne Paris Cité Research Center, Obstetrical, Perinatal and Pediatric Epidemiology Team, 53 avenue de l’observatoire, 75014 Paris, France; 4 Unité d’épidémiologie clinique, CIC-EC 1426, Assistance Publique-Hôpitaux de Paris, Hôpital Robert Debré, 48 boulevard Sérurier, 75019 Paris, France; 5 Inserm, U1123, ECEVE, 10 avenue de Verdun, 75010 Paris, France; 6 Inserm, U1138, Equipe 22, Sorbonne Université, Université Paris Descartes, 75006 Paris, France; 7 Service de néonatalogie, Hôpitaux universitaires de Genève, 32 boulevard de la Cluse, 1205 Genève, Switzerland; 8 Inserm, U1141, Hôpital Robert Debré, 48 boulevard Sérurier, 75019 Paris, France; 9 Service d’Accueil des Urgences Pédiatriques, Assistance Publique-Hôpitaux de Paris, Hôpital Robert Debré, 48 boulevard Sérurier, 75019 Paris, France

**Keywords:** Antenatal betamethasone, Antenatal corticosteroids, Preterm delivery, Preterm birth, Respiratory distress syndrome, Non-inferiority

## Abstract

**Background:**

Although antenatal betamethasone is recommended worldwide for women at risk of preterm delivery, concerns persist regarding the long-term effects associated with this treatment. Indeed, adverse events, mainly dose-related, have been reported. The current recommended dose of antenatal betamethasone directly derives from sheep experiments performed in the late 60’s and has not been challenged in 45 years. Therefore, randomized trials evaluating novel dose regimens are urgently needed.

**Methods:**

A randomised, double blind, placebo-controlled, non-inferiority trial will be performed in 37 French level 3 maternity units. Women with a singleton pregnancy at risk of preterm delivery before 32 weeks of gestation having already received a first 11.4 mg injection of betamethasone will be randomised to receive either a second injection of 11.4 mg betamethasone (full dose arm) or placebo (half dose arm) administered intramuscularly 24 h after the first injection. The primary binary outcome will be the occurrence of severe respiratory distress syndrome (RDS), defined as the need for exogenous intra-tracheal surfactant in the first 48 h of life. Considering that 20% of the pregnant women receiving the full dose regimen would have a neonate with severe RDS, 1571 patients in each treatment group are required to show that the half dose regimen is not inferior to the full dose, that is the difference in severe RDS rate do not exceed 4% (corresponding to a Relative Risk of 20%), with a 1-sided 2.5% type-1 error and a 80% power. Interim analyses will be done after every 300 neonates who reach the primary outcome on the basis of intention-to-treat, using a group-sequential non-inferiority design.

**Discussion:**

If the 50% reduced antenatal betamethasone dose is shown to be non-inferior to the full dose to prevent severe RDS associated with preterm birth, then it should be used consistently in women at risk of preterm delivery and would be of great importance to their children.

**Trial registration:**

ClinicalTrials.gov identifier: NCT 02897076 (registration date 09/13/2016).

**Electronic supplementary material:**

The online version of this article (10.1186/s12884-019-2206-x) contains supplementary material, which is available to authorized users.

## Background

### Benefits of antenatal corticosteroids

The beneficial pulmonary effects of antenatal corticosteroids (ACS) were originally discovered in sheep in the late 60’s when Liggins noticed the partial aeration of the lungs of preterm lambs born after ACS therapy [1]. In the first randomised trial evaluating the maternal administration of corticosteroids to induce surfactant protein synthesis and accelerate fetal lung maturity in preterm neonates, Liggins and Howie showed, in comparison with placebo, lower rates of respiratory distress syndrome (RDS) in newborns whose mothers had received 2 injections of 12 mg betamethasone 24 h apart [2], a dose directly extrapolated from their sheep studies. Since then, these results have been confirmed in many trials, with no change in or even further exploration of the best regimen to use. In a meta-analysis published in 1995, it was shown that ACS administration was not only beneficial to the lungs but also to the brain and intestinal tract of preterm infants [3]. ACS therapy was consequently adopted worldwide to prevent neonatal complications associated with preterm birth [4–7]. The latest meta-analysis by the Cochrane collaboration in 2017 demonstrated that a single course of ACS in singleton pregnancies at risk for preterm delivery was associated with a 38% reduction in RDS, a 48% reduction in intraventricular hemorrhage (IVH), and a 50% reduction in necrotizing enterocolitis (NEC), resulting in an overall 25% reduction in neonatal deaths [8].

### Concerns about antenatal corticosteroids

Despite short-term benefits, concerns persist regarding the long-term effects associated with ACS. Indeed, extensive animal studies have simultaneously revealed the impact of ACS on the programming of many fetal tissues and organs [9–11]. Across species, ACS administration results in alterations of the hypothalamic-pituitary-adrenal axis [12–17], in abnormal metabolism [16, 18, 19], in hypertension [16, 20], and in delayed myelination within the central nervous system [21–23]. In humans, because most clinical trials were performed in the 70’s, 80’s and early 90’s, before early childhood assessment of perinatal interventions became standard of good research practices, long term consequences of a single course of ACS have been poorly investigated. While neurodevelopmental adverse consequences have not been reported in children, some evidence exists regarding the interaction between betamethasone and brain maturation in rodents [24, 25], suggesting that lowering the dose of antenatal betamethasone may be less detrimental for the developing brain. Finally, a trend toward insulin resistance at 30 years of age has also been reported [26].

### Dose-related effects of antenatal corticosteroids

Furthermore, results from animal and human studies have shown that these side-effects could be dose-related. Indeed, studies in sheep [27], rabbits [28], mice [29] and rhesus monkeys [30] have demonstrated that repeated ACS courses were associated with alterations in fetal growth, findings confirmed in several large retrospective human studies [31–34] and in randomised trials comparing single and multiple courses [35–39]. Similarly, a trend toward increased rates of cerebral palsy has been reported at 2 years of age in children born after 34 weeks of gestation who received four or more full courses of betamethasone [40], but not half courses [41]. In a subgroup of children finally born at term, repeated antenatal betamethasone was associated with increased rates of neurosensory disabilities at 5 years [42]. Conversely, follow-up studies comparing single and multiple courses of betamethasone at 5 and 6–8 years of age have not shown differences in the rates of severe disabilities [43, 44] or in body composition, insulin sensitivity, ambulatory blood pressure or renal function [43, 44]. These results suggest that the long-term impact of ACS is probably underexplored and possibly underestimated.

### Dose reduction of antenatal corticosteroids

Clinical trials testing dose regimens other than 12 mg betamethasone twice 24 h apart have never been performed. However, sheep studies indicate that a 50% dose reduction is as effective to induce lung maturation as a full dose [45, 46]. In this context, because dosing of ACS has not been questioned in clinical trials for more than 40 years, the Cochrane collaboration concluded that evaluation of novel antenatal corticosteroid regimens was urgently needed [47].

### Aims of the trial

The primary aim of this non-inferiority 1:1 randomised trial is to determine whether a 50% reduction of the antenatal betamethasone dose given to women at risk of very preterm delivery is not inferior to a full dose to prevent severe RDS associated with preterm birth. We made the alternative hypothesis that the difference in failure rate between the reduced-dose and the full-dose arm does not exceed 4% (corresponding to a Relative Risk of 1.20).

The secondary aims are to compare other neonatal complications between the 50% reduced and the full antenatal betamethasone dose regimen.

## Methods/design

### Study design

Randomised, multicenter, double blind, placebo-controlled, group-sequential non-inferiority trial. The trial protocol (v 5.0, November 2018) adhered to the Standard Protocol Items: Recommendations for Interventional Trials (SPIRIT) 2013 Statement for protocols of clinical trials [48].

### Setting

Thirty-seven French level 3 maternity units will be involved (Additional file 1).

### Inclusion criteria

Women are eligible for the trial if they fulfill the all following criteria:

- Age ≥ 18 years
- Singleton pregnancy
- First betamethasone injection already performed
- Gestational age < 32 weeks at first betamethasone injection
- Informed consent form has been obtained by the investigating obstetrician or mid-wife

### Exclusion criteria

Women are not eligible for the trial if they fulfill one of the following criteria:

- They had already received a full course of betamethasone.
- The first injection has been given by the intravascular route
- In case of preterm labor:
  - ○ Cervical dilatation at or greater than 4 cm, or
  - ○ Ultrasonographic cervical length at or greater than 20 mm
- Any chromosomal aberrations and/or major fetal malformations
- Poor understanding of the French language

### Study interventions

Women of the standard full dose group will receive a second intramuscular injection of 11.4 mg of betamethasone (Celestene Chronodose, MSD France) 24 h after the first injection of betamethasone.

Women of the experimental half dose group will receive a second intramuscular injection of serum saline 24 h after the first injection of betamethasone.

Women of both groups justifying a rescue course of ACS are allocated a “rescue course treatment pack” from the same treatment group. All others concomitant care and interventions are permitted during the trial and none are prohibited.

### Study procedures (Fig. 1)

#### Recruitment

The trial information sheet is given to all eligible women after the first injection of betamethasone. They will be counseled by a member of the research team and encouraged to discuss the study with their family in the 24 h interval between the 2 injections and before the written informed signed consent is sought.

**Fig. 1:**
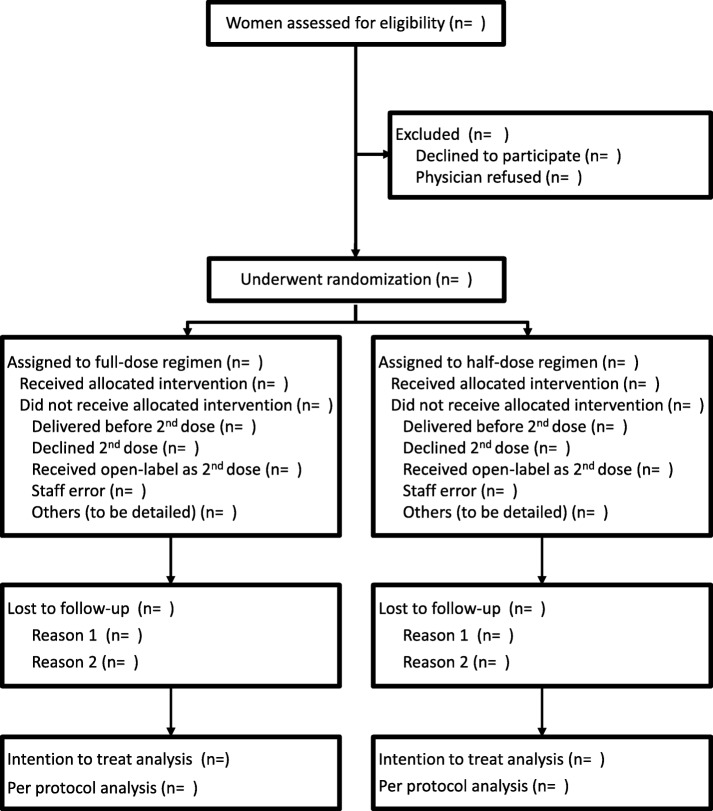
Flow chart of the trial

#### Allocation of treatment and blinding

After obtaining maternal consent, randomisation is performed just before the second injection, using a web-based application and a secured access, in a one-to-one ratio to the full or half dose groups according to a computer-generated list of randomly permuted blocks. The randomisation is stratified by maternity units and gestational age (before or after 28 weeks). The randomisation list is centrally computer-generated and a web-based application ensures proper allocation concealment. The allocation sequence is not available to any member of the research team until the database will be completed and locked. Patients, care providers and outcome assessors are masked to assignment. A study number is allocated to the woman corresponding to a treatment pack, each of which looks identical and contains two opaque study-labeled vials.

#### Unblinding procedures

Unblinding will be requested for any reason considered essential by the investigating doctor by calling upon:

- the DRCD in a situation other than an emergency during the work day and during working hours, addressed to the DRCD’s project referent.
- the poison centre of Fernand Widal Hospital, in the case of an emergency (see emergency situations requiring unblinding), on weekends, bank holidays, when the DRCD is closed and when unblinding cannot be carried out at the DRCD

#### Women follow-up

Women will be followed from randomisation to delivery.

#### Neonatal follow-up

Follow-up visit on Day1, 48 h after birth, Day2 to Day7, and Day28 will be performed by the neonatologists in the neonatology units for preterm neonates and on Day 1 and 48 h after birth by neonatologists in the postpartum units for full term neonates. During these visits, neonatologists will assess vital and ventilation parameters of the newborns, the primary and secondary outcome measures. The last research visit will take place in the postpartum units the day of hospital discharge for full term neonates and in the neonatology units not after 37 weeks of postmenstrual age (PMA) for neonates born preterm. At this visit, secondary outcome measures will be assessed by the neonatologists.

#### Long-term children follow-up

Neurodevelopmental assessment of children by certified neuropsychologists is planned at 3 years of age, but is not part of the present protocol.

### Outcome measures

#### Primary outcome measure

The primary assessment criterion is severe respiratory distress syndrome (RDS), defined as need for exogenous intra-tracheal surfactant within the first 48 h of life. It is considered as a binary endpoint: failure in case of severe RDS, or not failure.

#### Secondary outcome measures

The secondary assessment criteria will be measured during the neonatal period until hospital discharge for children born at term and not later than 37 weeks of PMA for the premature babies. They will include:

- Respiratory distress syndrome defined within the first 72 h as the use of continuous positive airway pressure and/or supplemental oxygen during at least 24 h, or the use of mechanical ventilation.
- Transient tachypnea of the newborn defined as a need for continuous positive airway pressure and/or supplemental oxygen, resolving within 24 h.
- Outcomes related to the severity of RDS:
  - ○ Highest appropriate fractional inspired oxygen (FiO2),
  - ○ Maximum appropriate Mean Airway Pressure (MAP),
  - ○ Use and duration of mechanical ventilation,
  - ○ Use and duration of oxygen therapy,
  - ○ Need for oxygen therapy after 36 weeks post conception.
- Outcomes related to betamethasone impact on other prematurity-induced complications:
  - ○ Neonatal death before discharge*,
  - ○ Admission to neonatal intensive care unit,
  - ○ Use of inotropic support including dopamine, epinephrine, dobutamine, and norepinephrine,
  - ○ Pneumothorax,
  - ○ Patent ductus arteriosus requiring either medical or surgical treatment,
  - ○ Bronchopulmonary dysplasia at 36 weeks of PMA (need for continuous positive airway pressure or supplemental oxygen, or mechanical ventilation),
  - ○ NEC and grade according to Bell classification [49],*
  - ○ IVH and grade according to the Papile classification [50],*
  - ○ Cystic periventricular leukomalacia,
  - ○ Use of postnatal steroids (either inhaled or systemic),
  - ○ Retinopathy of prematurity requiring anti-VEGF treatment or laser*,
  - ○ Length of hospital stay before the first discharge home,
  - ○ Survival without severe RDS, IVH grade 3 and 4, NEC grade ≥ 2, or retinopathy of prematurity treated by anti-VEGF or laser.
- Outcomes related to potential adverse events of betamethasone
  - ○ Birth weight at birth,
  - ○ Head circumference at birth,
  - ○ Body length at birth,
  - ○ Suspected or confirmed early onset sepsis treated using antibiotics during 7 days,
  - ○ Hypoglycaemia requiring oral or IV glucose administration or glucagon within 7 days.

Outcomes marked with an asterisk * are the four *safety outcomes* that will be monitored at each interim analysis.

### Data collection and management

Follow-up data will be collected by trained clinical research technicians on an electronical case-report-form (eCRF). To avoid women lost to follow-up, they will track women deliveries, especially when taking place outside the investigation centres. eCRFs shall be periodically cross-checked for completeness. A data management plan will be written and follow during all the data management and analysis process.

### Confidentiality and data handling

Data will be handled according to the French law. The eCRFs will be hosted by a service provider into a secured electronic system via a web navigator and protected by an individual password for each investigator and clinical research technician. Participant’s identifying information will be replaced by an unrelated sequence of characters to ensure confidentiality. The steering committee will have access to the full trial dataset. The trial database file will be stored for 15 years. The sponsor is the owner of the data.

### Statistical analysis

#### Sample size

To study the non-inferiority of the 50% reduced betamethasone dose regimen, we will test the alternative hypothesis that the difference in failure rate between the half-dose and the full-dose arm do not exceed 4% (corresponding to a Relative Risk of 1.20). This non-inferiority margin has been obtained through a consensus between the investigators of the GROG, neonatologists and the methodologists of the study, considering that a 4% difference is the smallest value that would be clinically relevant between arms and correspond to the preservation of a 70% of the effects of the full dose betamethasone regimen over placebo. Indeed, in the French Epipage2 study (2011) [51], 62% of the neonates exposed to antenatal betamethasone and born before 32 weeks of gestation from a singleton pregnancy received exogenous surfactant. Assuming that 33% (conservative hypothesis) of the randomised women will indeed deliver before 32 weeks, we estimated that 20% of the included pregnant women receiving the full betamethasone dose regimen would have a neonate with severe RDS, defined as the need for exogenous intra-tracheal surfactant. According to the literature, ACS is responsible for an average relative risk of RDS of 0.66 (95% CI 0.56 to 0.77), compared to placebo [8, 52]. Assuming a prevalence of severe RDS of 20% in the full dose betamethasone regimen, to preserve 67% of the upper bound for the historical difference between full dose and placebo (i.e. 0.67 x (0.20–0.20/0.77)) gave a margin of 4% (or expressed as Relative Risk (20 + 4) / 20 = 1.20). Thus, 1571 patients in each treatment group are required to test the non-inferiority hypothesis, with a 1-sided type-1 error of 0.025, a power of 0.80, and a non-inferiority margin equal to 4% [53].

#### Analysis population

The primary non-inferiority statistical analysis will be performed according to both the intention-to-treat and per protocol principle, as it is recommended for non-inferiority trials [54]. The intention-to-treat population will included all randomised patients according to the treatment group where they have been randomly assigned, regardless of what treatment, if any, they received. The per protocol (PP) analysis will included only the participants who fulfill the protocol in terms of eligibility, interventions and outcome assessment: the women will be excluded from this analysis if they did not fulfill the eligibility criteria, if they did not receive any intervention after randomisation, if they received the intervention of the opposite arm as first and/or rescue course, and if the intervention was overdosed or intravenous. Women who received the first course as they were randomised but who received an incomplete rescue course will be analyzed in their randomisation arm.

#### Interim analysis

To address the ethical concerns of (i) studying a high-risk population (pregnant women and preterm neonates), (ii) the potential increased rates of babies with severe RDS due to the reduction of the betamethasone dose, (iii) the anticipated length of time of inclusion (30 months), (iv) the number of infants planned for a fixed analysis (*n* = 3142) and (v) the primary endpoint measured in the first 48 h of life, a sequential data analysis method will be used, allowing to provide stopping rules. Analyses will be done after every 300 neonates who reach the primary outcome on the basis of intention to treat. The trial may be stopped for the following reasons:

i. Inferiority of the experimental group on the primary outcome,
ii. Non-inferiority of the experimental group on the primary outcome,

Finally, a maximum of 11 analyses is planned.

For the primary outcome, a non-inferiority sequential design with alpha and beta spending functions will be used to control the first type and second type error, respectively. For the estimation of the boundary curves, we chose a monotone function proposed by *Kim and DeMets* and generalized by *Jennison and Turnbull* [55, 56]. Critical values of the boundary curves are defined for each interim analysis. At each interim analysis, the maximum likelihood estimator of the difference of treatment failure rates between full-dose and half-dose will be compared to these critical values and the necessity to stop the trial will be checked.

In addition to that sequential primary analysis, the rate and percentage of the four safety outcomes (marked above with an *: neonatal death, IVH grade 3 and 4, NEC grade ≥ 2, and retinopathy of prematurity treated by anti-VEGF or laser) will be estimated in order to detect a potential increasing in the experimental group. Those estimation will be made on the overall population, and by gestational age at birth (born before 28 weeks, between 28 and 32 weeks, and after 32 weeks), as expected prevalence are different from one subgroup to the other.

#### Final analyses

Data analysis and reporting will follow the CONSORT guidelines for non-inferiority randomised controlled trials [57]. The two groups will be compared for women’s and neonate’s characteristics. Qualitative variables will be summarised by numbers and percentages of patients in each treatment group.

The final primary non-inferiority statistical analysis will be conducted on all the neonates enrolled in the trial, including those who did not participate in the interim analysis. The difference between the failure rates observed in both arms along with its 2-sided confidence interval will be estimated. The final boundary for difference will be compared to the critical value corresponding to the number of women finally included, to claim or not the non-inferiority. A figure showing confidence intervals and the margin of non-inferiority will be used to summarize the result on the primary outcome.

The analyses on the other pre-specified secondary outcomes will consist in estimations and comparisons between the two arms. The 95% confidence interval for the difference between arms will be constructed. The χ2 or Fisher’s exact test for categorical variables and Student or Mann-Whitney-Wilcoxon test for continuous variables will be used to compare the full-dose and the half-dose regimens, according to the validity conditions. All these statistical tests will be two-sided and the level of statistical significance will be set at 5% (2-sided).

#### Subgroup analyses

Planned subgroup analyses include:

- Gestational age at birth (born before 28 weeks, between 28 and 32 weeks, and after 32 weeks)
- Gender of the newborn

For those subgroups, we will repeat:

- The primary analysis, using the confidence interval for the difference between full-dose and half-dose.
- The following secondary analysis: death, IVH grade 3 and 4, NEC grade ≥ 2, or retinopathy of prematurity treated by anti-VEGF or laser, survival without severe RDS, IVH grade 3 and 4, NEC grade ≥ 2, or retinopathy of prematurity treated by anti-VEGF or laser, using 2-sided tests, as stated above.

For both those analyses, we will use the Holm-Bonferroni method to adjust for multiplicity of analyses [58].

Statistical analyses will be performed with SAS (V.9.4; SAS institute) and R (V 3.4.2) software.

### Trial steering committee

A trial steering committee will include the coordinating investigator (TS), the scientific director (OB), the biostatistician (MU), the methodologists (CAi and CAs), and the representatives for the sponsor and for the unit in charge for the data collection and management. They will be responsible for the organization and the coordination of the trial. They will meet on a quarterly basis to review the progress of the trial.

### Safety monitoring

The trial safety will be evaluated by an independent Data Safety Monitoring Board (DSMB) at each interim analysis or when additional analyses will be requested by the sponsor or the steering committee. The DSMB will include experts in or representatives of the fields of obstetrics, neonatology, and clinical trials methodology. At the first time they met, the DSMB will validate that the methodology is compatible with the safety of the participants. Prior to each DSMB meeting, they will be provided a complete list of all adverse events, and a statistical report including description of the population and results of the interim analysis as described above. At each meeting, the DSMB may give the advice to temporarily or definitely stop the trial if in their opinion there is an unexpected or unacceptable risk for the women or the newborn, or if the interim analysis suggests the non-inferiority or futility.

### Ethics

The statistical plan has been written before the starting of the trial and approved by the steering committee, the sponsor, the French Agence National pour la Sécurité du Médicament (ANSM) and the DSMB. All modifications will be subject to the approval of these entities.

### Dissemination policy

The steering committee will determine the plan for dissemination policy. Authorship for manuscripts submitted for publication will follow the criteria defined by the International Committee of Medical Journal Editors.

## Discussion

Administration of antenatal betamethasone to women at risk of preterm delivery leads to substantial benefits for babies born preterm. Although this treatment is widely used and recommended worldwide, concerns persist regarding its long term effects because adverse events, mainly dose-related, have been reported. Because the current recommended dose of antenatal betamethasone directly derives from sheep experiments performed in the late 60’s and has not been challenged in 45 years, large randomised trials evaluating novel dose regimens are urgently needed. If a 50% reduced antenatal betamethasone dose is shown non inferior to a full dose to prevent the neonatal complications associated with preterm birth then it should be used consistently in women at risk of preterm delivery and it would be of great importance to their children.

## Additional file

Additional file 1: Table 1: List of the participating centers. (DOCX 36 kb)

## Abbreviations

ACS: Antenatal corticosteroids
eCRF: Electronical case report form
FiO2: Fractional inspired oxygen
IVH: Intraventricular hemorrhage
MAP: Maximum airway pressure
NEC: Necroziting enterocolitis
PMA: Post menstrual age
RDS: Respiratory distress syndrome
