# Supplementary material for: Full versus half dose of antenatal betamethasone to prevent severe neonatal respiratory distress syndrome associated with preterm birth: study protocol for a randomised, multicenter, double blind, placebo-controlled, non-inferiority trial (BETADOSE)
Source: BMC Pregnancy Childbirth. 2019 Feb 12;19:67. doi: 10.1186/s12884-019-2206-x (PMC6373166; doi:10.1186/s12884-019-2206-x)
Supplement: Supplementary file 1 — Table 1: List of the participating centers. (DOCX 36 kb) [file 12884_2019_2206_MOESM1_ESM.docx]

**Table 1:** List of the participating centers

| **Hospital Name** |
| --- |
| CHRU de Lille |
| CH de Valenciennes |
| CHU d’Amiens |
| CHU de Caen |
| CHU de Cherbourg |
| CHU de Nantes |
| CHU d’Angers |
| CH de Vannes |
| CHU de Rennes |
| CHU de Strasbourg |
| Maternité Régionale de Nancy |
| CHU de Tours |
| CHR d’Orléans |
| CHU de Limoges |
| CHU de Bordeaux |
| CH de Pau |
| CHU de Toulouse |
| CHU de Clermont-Ferrand |
| CHU de Saint Etienne |
| Lyon, Hôpital Mère Enfant |
| CHU de Grenoble |
| CHU de Nîmes |
| CHU de Montpellier |
| APHM, La Conception, Marseille |
| APHM, Hôpital Nord, Marseille |
| APHP, Robert Debré, Paris |
| APHP, Port-Royal, Paris |
| APHP, Trousseau, Paris |
| APHP, Necker, Paris |
| APHP, Antoine Béclère, Clamart |
| APHP, Bicêtre, Le Kremlin-Bicêtre |
| APHP, Louis Mourier, Colombes |
| CH Delafontaine, Saint-Denis |
| CHI de Créteil |
| CHI de Poissy |
| CH de Corbeil Essonne |
| CH de Pontoise |
